# Parenting and Love Styles: A Cross-National Study of Angolan and Italian Emerging Adults

**DOI:** 10.3390/bs16040538

**Published:** 2026-04-03

**Authors:** Alessandra Fermani, Carla Canestrari, Ramona Bongelli, Gonzalo Del Moral Arroyo, Manuel Teresi

**Affiliations:** 1 Department of Education, Cultural Heritage and Tourism, University of Macerata, 62100 Macerata, Italy; alessandra.fermani@unimc.it (A.F.); ramona.bongelli@unimc.it (R.B.); manuel.teresi@unimc.it (M.T.); 2 Department of Education and Social Psychology, University of Sevilla “Pablo de Olavide”, 41089 Dos Hermanas, Spain; gmorar@upo.es

**Keywords:** love styles, parental attachment, emerging adulthood, Italy, Angola

## Abstract

The present study examined how parental attachment and cultural background shape love styles in emerging adulthood. Drawing on attachment theory and cross-cultural perspectives, we investigated whether gender, attachment to mother and father, and nationality (Italian vs. Angolan) predicted the development of love styles in 370 young adults. Participants completed validated measures of parental attachment (IPPA) and love attitudes (LAS). Hierarchical regression analyses revealed a differentiated pattern across love styles. Emotionally detached relational styles (Ludus) were significantly predicted by gender, paternal attachment, and nationality, with higher levels reported by men, those with lower paternal attachment, and Angolan participants. While Storge showed no significant associations, passionate love expression (Eros) was robustly predicted by nationality, with Italian participants reporting higher levels of passion in love. Results suggest that while paternal attachment serves as a critical developmental anchor in preventing Ludus, the cultural macrosystem remains the primary architect of Eros. These findings call for culturally attuned clinical and educational interventions that differentiate between early relational deficits and normative cultural variations in intimacy.

## 1. Introduction

Interest in studying parental attachment and its impact on emerging adults’ romantic relationships has grown in recent years. Understanding the feelings of emerging adults concerning attachment figures is important because these relationships will have an impact on their future well-being and potential parenthood (Fermani et al., 2019; Galinha et al., 2014). Love and attachment are among the most universal human experiences; however, their meanings and expressions are profoundly shaped by culture.

Attachment theory, proposed by John Bowlby and empirically expanded by Ainsworth et al.’s (2015) research team, posits that early caregiving experiences shape internal working models that guide expectations and behaviors in relationships across the lifespan. Four main attachment styles have been identified: secure, anxious, avoidant, and disorganized. Secure attachment is characterized by a positive view of the self and others, comfort with intimacy, and effective emotional regulation. Anxious attachment involves a negative self-view, fear of abandonment, and heightened reassurance-seeking. Avoidant attachment is marked by discomfort with closeness, emotional suppression, and a preference for self-reliance. Disorganized attachment reflects contradictory or dysregulated relational strategies, often linked to unresolved fear or trauma. These patterns significantly influence romantic functioning: secure attachment is associated with trust and relationship satisfaction, while insecure patterns—encompassing anxious, avoidant, and disorganized styles—are characterized by emotional distancing, relational instability, and a heightened fear of abandonment (Mikulincer & Shaver, 2016).

Attili (2017) reaffirmed the link between attachment theory and Lee’s (1973) love styles. Psychologically, Eros reflects passionate attraction and emotional intensity; Agape represents altruistic, self-giving love grounded in care and commitment; Ludus denotes game-playing, emotionally detached love; Pragma refers to rational, goal-oriented love based on compatibility and practical considerations; and Storge indicates affectionate, friendship-based love rooted in familiarity and stability. Secure individuals tend to combine Eros and Agape, expressing both passion and caregiving, and show adaptive coping after breakups. Conversely, insecure attachment is associated with defensive strategies such as emotional distancing (Lenzi et al., 2013), compulsive partner seeking, jealousy, dependence, or avoidance of intimacy, reflecting difficulties in trust, stress regulation, and emotional co-regulation in romantic relationships.

Once acquired in childhood, these attachment styles tend to remain relatively stable lifelong, as attachment figures shift from parents to partners during emerging adulthood (Bowlby, 1969/1982; Wallin, 2007). The choice of partner and the way individuals behave are influenced by the quality of the bond established with parents during childhood (Momeñe et al., 2024).

In this framework, it is crucial to distinguish between maternal and paternal contributions. While maternal attachment is traditionally associated with emotional containment, the Paternal Activation Relationship Theory suggests that fathers specifically foster the child’s ability to navigate the social world and regulate risk (Dumont & Paquette, 2013). In emerging adulthood, a secure paternal bond may facilitate healthy relational exploration, whereas lower paternal attachment might lead individuals to adopt emotionally detached or game-playing strategies as a defense mechanism against intimacy (Eberwein & Gogineni, 2024).

Cross-cultural research consistently highlights the distinction between collectivist and individualist cultural orientations (Triandis, 1995, 2001). Collectivist cultures construe the self as interdependent, prioritizing social harmony and group goals, with personal decisions strongly shaped by family expectations and in-group obligations (Hofstede, 2001; Triandis, 1995). In contrast, individualist cultures emphasize autonomy and personal achievement, encouraging individuals to act as per internal preferences even when they diverge from social norms (Kagitçibasi, 2005; Markus & Kitayama, 1991). Although these orientations exist along a continuum, they have meaningful implications for attachment and romantic development in emerging adulthood (Kagitçibasi, 2007). However, most research on attachment and love styles has been conducted exclusively on WEIRD (Western, Educated, Industrialized, Rich, and Democratic) populations (e.g., Hendrick & Hendrick, 1986; Henrich et al., 2010; Lee, 1973), raising questions about the universality of the links between parental attachment and romantic love. Particularly, little is known about how culturally distinct parental roles in collectivist versus individualist contexts may shape emotional development and adult intimacy (Markus & Kitayama, 1991; Triandis, 1995).

Within the cultural framework, emerging adults’ romantic experiences differ systematically across societies, reflecting broader norms concerning autonomy and family interdependence. In collectivist settings, as in many African countries, family obligations remain a central influence, and parental approval plays a salient role in partner selection. In more individualist societies, such as Italy, romantic choices primarily reflect personal preferences, emotional independence, and self-determination (Arnett, 2007). Angola represents a predominantly collectivist context where socialization emphasizes interdependence, respect for elders, and community support, and decisions regarding romantic relationships are strongly shaped by familial expectations. Consequently, attachment to parents continues to guide emotional and relational choices into emerging adulthood (Bejanyan et al., 2015; Buunk et al., 2010; Koumba Maguena et al., 2025). Italy, while culturally family-oriented, aligns more closely with Western individualism, and the negotiation of romantic relationships is generally guided by personal fulfillment and emotional autonomy. In this context, parental attachment remains a significant predictor of romantic competence and relationship satisfaction in emerging adulthood (Kumar & Mattanah, 2016). Comparing Angola and Italy provides a theoretically meaningful basis for examining how parental attachment influences romantic decision-making across diverse cultural environments. Angola experienced a prolonged civil war (1975–2002) following independence from Portugal, a conflict that profoundly shaped the country’s social fabric and family structures. Decades of armed violence, forced displacement, economic instability, and loss exposed large segments of the populace to chronic stress and trauma. Research in post-war Angola has documented high levels of psychological distress and trauma exposure among civilians, including caregivers (Wessells & Monteiro, 2001). Extensive literature on political violence further reveals that chronic insecurity and trauma can influence parenting practices, sometimes fostering more emotionally restrained or authoritarian styles as adaptive responses to instability (Alleyne-Green et al., 2019; Barber, 1999). In post-conflict contexts, caregivers may prioritize discipline, protection, and material provision over emotional expressiveness—not necessarily due to a lack of affection, but as a culturally and historically shaped strategy aimed at ensuring children’s safety and resilience. Acknowledging the impact of Angola’s civil war, therefore, contributes to a more balanced interpretation of emotionally distant parental roles in the broader socio-historical context. Specifically, in collectivist contexts like Angola, parental attachment is expected to exert a stronger and more direct influence on partner choice (Buunk et al., 2010), whereas in individualistic contexts such as Italy, its influence may be more indirect, mediated by the development of autonomy and personal realization (Kumar & Mattanah, 2016). Concurrently, recent socioeconomic changes, such as industrialization and globalization, have led many Angolan emerging adults to adopt lifestyle patterns typical of Western emerging adulthood; nevertheless, empirical research on romantic relationships in Angola remains limited. Cross-cultural evidence suggests that romantic relations are shaped by socioeconomic factors, gender norms, and family expectations, with love choices embedded in broader kinship and economic dynamics (Da Silva, 2013; Mazzucato et al., 2015; Pinho et al., 2016; Voges et al., 2019), further underscoring the need for culturally grounded comparative research.

### The Present Research

The present study addresses a gap in the literature by moving beyond WEIRD populations, which have historically dominated attachment research. By comparing a Southern European context (Italy) with a Lusophone African context (Angola), this research tests the universality of attachment-love links. It aims to examine the predictors of romantic love styles in emerging adulthood in a cross-cultural framework grounded in attachment theory and cross-cultural psychology (Bowlby, 1969/1982; Hazan & Shaver, 1987). Besides attachment and cultural context, gender differences have been widely documented in romantic orientations. Previous research suggests that men and women may differ in the extent to which they endorse emotionally detached versus emotionally involved approaches to romantic relationships, particularly during emerging adulthood (Fehr, 1994; Hatfield & Rapson, 1993). Therefore, gender was considered a theoretically relevant individual-level predictor in the present study. Specifically, it is hypothesized that (Hp1) gender will significantly predict romantic love styles, as gendered socialization processes shape emotional expression, intimacy regulation, and relational orientations (Cross & Madson, 1997; Del Giudice, 2011; Rothbaum et al., 2000). Building upon the principle of developmental continuity, it is further hypothesized that (Hp2) parental attachment to both mother and father will significantly predict romantic love styles, with the quality of early relational bonds providing the foundational internal working models for adult romantic orientations (Hazan & Shaver, 1987; Fermani et al., 2019, 2020). Finally, drawing on the premise that models of the self and intimacy are culturally situated, it is hypothesized that (Hp3) cultural context (i.e., nationality) will significantly explain additional variance in romantic love styles beyond the effects of gender and individual attachment histories, reflecting the influence of diverse cultural scripts and relational norms on the expression of intimacy (Kagitçibasi, 2007; Markus & Kitayama, 1991; Triandis, 1995). By testing these associations, the study seeks to elucidate how broader societal frameworks and early relational experiences uniquely modulate the expression of romantic love during the transition to adulthood.

## 2. Materials and Methods

### 2.1. Participants

Three hundred and seventy participants were recruited from two distinct geographical settings—Italy and Angola—to facilitate a cross-cultural comparison. Selection was based primarily on age, which was strictly maintained between 19 and 29, consistent with the developmental stage known as emerging adulthood (Arnett, 2007).

The Italian subsample comprised 182 participants, while the Angolan subsample numbered 188. The mean age for the entire sample was 26.61 years (SD = 2.47). Regarding gender, the total sample comprised 202 women (Italian: 95, Angolan: 107) and 168 men (Italian: 87, Angolan: 81). Inclusion criteria required participants to be residents in their respective countries and to possess native-level comprehension of the language used in the survey (Italian or Portuguese). Participants reporting clinical conditions that could interfere with the study were excluded. Recruitment was through convenience sampling in collaboration with local academic institutions. A research assistant approached potential participants (including students and the general public) to seek their voluntary participation. The procedure involved a paper-and-pencil questionnaire, which took approximately 15 min to complete. As per the 1964 Declaration of Helsinki, anonymity was guaranteed. Upon completion, the participants were thanked and verbally debriefed.

### 2.2. Instruments

In this study, the shortened version of the Inventory of Parent and Peer Attachment (12 items) (IPPA; Armsden & Greenberg, 1987; Raja et al., 1992) was administered to assess emerging adults’ perceived relationship quality with their parents. The scale has been adapted in Italian or Portuguese, based on the country in which the survey was completed. Items were rated on a six-point Likert scale ranging from 1 (completely untrue) to 6 (completely true). Example items include: “My father/mother respects my feelings” (trust), “I talk to my father/mother about my problems and worries” (communication), and “My father/mother does not care much about me” (reverse-scored; closeness). For the purposes of the analyses, we computed separate global attachment scores for the mother (α = 0.76) and the father (α = 0.87) by averaging the corresponding sub-dimensions (trust, communication, and closeness). Only these composite indices were used to capture participants’ overall attachment to each parental figure.

Further, we employed the LAS (Hendrick & Hendrick, 1986), a 42-item questionnaire designed to assess individuals’ attitudes toward love. Hendrick and Hendrick (1986, pp. 400–401) describe the six factors extracted, and recent literature (Li et al., 2026; Neto & Pinto, 2025) proposes the same conceptualization.

1. Eros: Strong physical preferences, early attraction, and intensity of emotion are attributes of erotic love, along with a strong commitment to the lover. For instance, an individual may feel immediate chemistry upon meeting a partner and rapidly develop a deep, passionate commitment.
2. Ludus: Love is characterized as a game-playing approach, often involving diverse partners and a lack of emotional intensity. In this style, the deception of a lover may be considered acceptable within certain relational limits, as ludic lovers tend to be wary of intimacy and prioritize maintaining control. This orientation often carries a manipulative quality, which may result in lower social desirability. However, ludic aspects—such as dating multiple partners simultaneously or avoiding emotional commitment—can be present in many romantic relationships, serving as a strategy to maintain autonomy.
3. Storge: This style reflects an inclination to merge love and friendship. There is no spark or fire in Storgic love; it is solid, down-to-earth, and presumably enduring. Storge represents love grounded in friendship and gradual attachment, as illustrated by two long-time friends whose relationship slowly evolves into a stable, affectionate partnership without intense passion.
4. Pragma: Rational calculation with a focus on desired attributes of the lover is central to pragmatic love. In fact, “love planning” might be an apt description (e.g., when someone evaluates a potential partner based on compatibility factors such as education, values, career stability, and long-term life goals).
5. Mania: Reading the items suggests that Mania is “symptom love,” based on the uncertainty of self and the lover. It may be most characteristic of adolescents, but examples of older manic lovers frequently occur (e.g., when an individual becomes obsessively preoccupied with their partner, fears abandonment, and frequently seeks reassurance about the relationship).
6. Agape: Lee (1973) did not find this style manifested fully in actual human beings. However, the factor results suggest that it is a viable style (e.g., when a person prioritizes their partner’s well-being above their own interests and offers unconditional support without the expectation of reciprocity).

The instrument captures attitudes directed toward one’s current, most recent, or hypothetical romantic partner, as well as general beliefs about love. Participants were instructed to respond with their current partner in mind; if they did not have a partner, they were asked to reflect on their most recent relationship. Those who had never been in love were instructed to provide the response they believed would best reflect their likely attitudes. Items are rated on a five-point Likert scale ranging from 1 (strongly agree) to 5 (strongly disagree). The scale consists of six subscales (seven items each), corresponding to distinct love styles: Eros (passionate love; e.g., “Our lovemaking is very intense and satisfying,” α = 0.83), Ludus (game-playing love; e.g., “I try to keep my lover a little uncertain about my commitment to him/her,” α = 0.62), Storge (friendship-based love; e.g., “The best kind of love grows out of a long friendship,” α = 0.61), Pragma (practical love; e.g., “I try to plan my life carefully before choosing a lover,” α = 0.75), Mania (possessive, dependent love; e.g., “I cannot relax if I suspect that my lover is with someone else,” α = 0.64), and Agape (altruistic love; e.g., “I am usually willing to sacrifice my own wishes to let my lover achieve his/hers,” α = 0.71).

### 2.3. Procedures

To address the research questions and test the hypotheses, descriptive and inferential statistical analyses were conducted using the Statistical Package for the Social Sciences (SPSS, version 28.0).

## 3. Results

Table 1 presents descriptive statistics and Pearson’s correlations for the total sample, while Table 2 reports means, standard deviations, and correlations calculated separately for the Italian and Angolan subsamples. Examination of the correlation patterns indicated that parental attachment variables were consistently associated with Ludus, Storge, and Eros across the two cultural contexts, whereas the remaining love styles did not show stable associations.

On this basis, hierarchical regression analyses were conducted focusing on these outcomes to examine whether gender, parental attachment (mother and father), and nationality predicted the love styles Ludus, Storge, and Eros, enabling us to retain a parsimonious and theoretically grounded model structure. Gender (coded 1 = female, 2 = male) was entered at Step 1, parental attachment variables at Step 2, and nationality (coded 1 = Italian, 2 = Angolan) at Step 3. In Step 1, gender significantly predicted Ludus (β = 0.12, *p* = 0.027; R^2^ = 0.013, F(1, 362) = 4.95, *p* = 0.027). At Step 2, the inclusion of mother and father attachment significantly increased the explained variance of the model, ∆R^2^ = 0.031, ∆F(3, 360) = 5.81, *p* = 0.003. In this model, gender (β = 0.130, *p* = 0.014) and father attachment (β = −0.173, *p* = 0.001) were significant predictors of Ludus, whereas mother attachment was not (β = −0.018, *p* = 0.737). At Step 3, nationality was added as a predictor and further increased the explained variance, ∆R^2^ = 0.069, ∆F(4, 359) = 28.09, *p* < 0.001. In this final model, gender (β = 0.138, *p* = 0.007), father attachment (β = −0.137, *p* = 0.008), and nationality (β = 0.266, *p* < 0.001) were significant predictors of Ludus. Mother attachment remained non-significant (β = −0.476, *p* = 0.634).

For Storge, Step 1 (gender) was not significant (β = −0.013, *p* = 0.799; R^2^ = 0.000, F(1, 362) = 0.07, *p* = 0.799). Adding mother and father attachment at Step 2 did not significantly improve the model, ∆R^2^ = 0.016, ∆F(3, 360) = 2.287, *p* = 0.058. At Step 3, the inclusion of nationality did not produce a significant increase in explained variance, ∆R^2^ = 0.000, ∆F(4, 359) = 0.066, *p* = 0.798. In the final model, none of the predictors (gender, mother attachment, father attachment, nationality) was statistically significant (all *p* > 0.05).

For Eros, Step 1 (gender) was not significant (β = −0.032, *p* = 0.539; R^2^ = 0.001, F(1, 362) = 0.38, *p* = 0.539). Step 2, which added mother and father attachment, also did not significantly improve the model, ∆R^2^ = 0.010, ∆F(3, 360) = 1.57, *p* = 0.209. However, the addition of nationality at Step 3 produced a substantial and significant increase in explained variance, ∆R^2^ = 0.200, ∆F(4, 359) = 90.87, *p* < 0.001. In the final model, nationality emerged as the only significant predictor (β = −0.452, *p* < 0.001), whereas gender (β = −0.057, *p* = 0.237), mother attachment (β = −0.000, *p* = 0.999), and father attachment (β = −0.032, *p* = 0.509) were not significant.

## 4. Discussion

The present study investigated the role of parental attachment and cultural background in shaping different love styles in emerging adulthood. Overall, the findings reveal a differentiated pattern, suggesting that early relational experiences and socio-cultural context contribute distinctly to how romantic orientations are expressed during this developmental phase. Specifically, while some love styles appear more sensitive to cultural scripts, others are more closely rooted in early relational bonds. The first relevant result concerns the role of paternal attachment in predicting Ludus. Lower levels of attachment to the father are associated with a higher endorsement of a more game-playing and emotionally detached approach to romantic relationships. This finding is broadly consistent with theoretical perspectives that emphasize the role of paternal relationships in encouraging exploration, autonomy, and the regulation of interpersonal risk. In this sense, weaker paternal attachment may be associated with greater emotional distance in romantic bonds, potentially fostering relational styles characterized by reduced commitment and a more playful orientation. Within this differentiated pattern, attachment to the mother did not emerge as a significant predictor of any of the love styles considered. Rather than suggesting a diminished role of maternal relationships, this result may indicate that early caregiving experiences do not exert a uniform influence across parental figures. Paternal and maternal bonds may contribute in partially distinct ways to later romantic orientations, a possibility that warrants further investigation. Gender also emerged as a significant predictor of Ludus, with men reporting higher levels than women. This pattern is consistent with previous literature suggesting that men are more likely to endorse less emotionally committed and more exploratory approaches to romantic involvement, particularly in emerging adulthood, a period marked by identity exploration and relational experimentation. Nationality further contributed to the prediction of Ludus, with Angolan participants reporting higher levels than Italian participants. This difference may reflect culturally shaped norms regarding emotional expression, relational commitment, and expectations in romantic relationships. As suggested by cross-cultural perspectives on intimacy and the self, cultural contexts provide shared meanings and scripts that influence how individuals approach closeness, autonomy, and emotional involvement (Kagitçibasi, 2007; Neto et al., 2000; Nsamenang, 2002). Conversely, Storge was not significantly associated with gender, parental attachment, or nationality. This absence of significant predictors suggests that companionate and friendship-based forms of love may be less directly rooted in early attachment experiences or broad cultural distinctions, and may instead develop more strongly through relational history, shared experiences, and gradual emotional bonding over time. In this sense, Storge may represent a dimension of romantic orientation shaped more by the unfolding of interpersonal relationships than by early family dynamics or macro-cultural factors. The most robust finding emerged for Eros, for which nationality was the only significant predictor. Italian participants reported higher levels of passionate love than Angolan participants, even after controlling for gender and parental attachment. This suggests that the experience and expression of intense romantic involvement may be particularly sensitive to culturally embedded models of intimacy, emotional expressiveness, and expectations surrounding romantic relationships. In line with cross-cultural research on relational norms, passionate love appears here to be more strongly shaped by socio-cultural context than by early attachment experiences alone. This distinction highlights a dual process in romantic development: while the relational style (e.g., the tendency toward Ludus) appears anchored in early paternal dynamics and internal working models, the emotional intensity and expression of passion (Eros) are primarily dictated by cultural scripts. This suggests that in contexts like Angola and Italy, cultural macrosystems may play a more prominent role than individual attachment histories in shaping the social expression of passionate love (Nsamenang, 2002). Taken together, these findings contribute to the literature by highlighting that parental attachment does not uniformly predict all dimensions of romantic orientation. It rather appears to be specifically linked to more emotionally distant relational styles, whereas cultural background plays a vital role in shaping the expression of passionate love. This pattern supports the idea that internal working models derived from early relationships operate within broader cultural frameworks that influence how intimacy is understood and expressed in emerging adulthood.

### 4.1. Practical Implications

From an applied perspective, these results may be relevant for professionals working with young adults in educational, clinical, and counseling settings. Greater awareness of the association between early paternal relationships and more emotionally detached relational styles may help practitioners better contextualize difficulties concerning intimacy, trust, and commitment. Concurrently, the strong role of cultural context in shaping passionate love underscores the importance of adopting culturally sensitive perspectives when addressing relationship expectations, emotional expression, and couple dynamics in diverse populations.

### 4.2. Limitations and Future Research Directions

Some limitations of the present study should be acknowledged. First, the cross-sectional nature of the design does not permit causal interpretations of the observed associations. Second, the reliance on self-report measures may have introduced shared method variance and potential social desirability effects. Third, participants were recruited through convenience sampling, which may limit the generalizability of the findings. Additionally, although the cross-cultural comparison represents a strength of the study, cultural differences were operationalized through nationality, which may not fully capture the complexity of cultural values, family norms, and relational expectations. Another aspect for consideration concerns the internal consistency of some dimensions of the love styles measure, which showed relatively modest alpha coefficients in the present sample. While these values were still within an acceptable range for exploratory cross-cultural research, they may partly reflect the challenges associated with administering and interpreting a scale translated into a different linguistic and cultural context from the one in which it was originally validated. Consequently, subtle differences in meaning, emotional connotations, or relational concepts may have influenced how certain items were understood. Future research would benefit from further cultural adaptation and validation work, including the refinement of translated items and additional psychometric testing, to ensure optimal reliability across contexts. This study’s exploratory nature represents a necessary step toward a more inclusive cross-cultural psychology. By mapping these preliminary associations in a previously understudied population, this work provides a foundation for future longitudinal studies to further clarify the causal pathways between paternal involvement and romantic development in non-Western contexts. It would be particularly valuable to include more nuanced cultural indicators (such as values related to family roles, emotional expression, and relational expectations) as well as potential mediating processes, including emotional regulation, relationship beliefs, and peer attachment.

### 4.3. Conclusions

The present study highlights the joint role of early relational experiences and cultural context in shaping romantic orientations in emerging adulthood. While paternal attachment appears to be specifically linked to more emotionally detached love styles, cultural background emerges as a crucial factor in the expression of passionate love. These findings reinforce the importance of integrating attachment and cultural perspectives to better understand the complexity of romantic development across diverse social contexts.

## Figures and Tables

**Table 1:** Means, standard deviations, and correlations among the main variables of the total sample.

	M(DS)	MA	FA	Eros	Ludus	Storge	Pragma	Mania	Agape
MA	4.60(0.81)	1	-	-	-	-	-	-	-
FA	3.98(1.09)	0.11 *	1	-	-	-	-	-	-
Eros	3.41(0.98)	0.01	0.09	1	-	-	-	-	-
Ludus	2.69(0.81)	−0.06	−0.16 **	−0.17 **	1	-	-	-	-
Storge	2.90(0.79)	−0.08	−0.10 ^a^	0.20 ***	0.24 ***	1	-	-	-
Pragma	2.84(0.89)	−0.06	−0.13 *	−0.09	0.41 ***	0.29 ***	1	-	-
Mania	2.96(0.76)	0.02	−0.09	0.27 ***	0.06	0.31 ***	0.17 **	1	-
Agape	3.12(0.81)	−0.05	0.06	0.50 ***	−0.14 *	0.23 ***	−0.11 *	0.29 ***	1

Note: *p* < 0.05 *; *p* < 0.01 **; *p* < 0.001 ***; ^a^ = 0.053; MA = Mother Attachment; FA = Father Attachment.

**Table 2:** Country-wise means, standard deviations, and correlations among the main variables of the study. Correlations relative to the Italian sample are reported below the diagonal and those pertaining to the Angolan sample above the diagonal.

	M(DS)	MA	FA	Eros	Ludus	Storge	Pragma	Mania	Agape
M(DS)	-	4.61(0.78)	3.84(1.11)	2.97(0.99)	2.91(0.79)	2.89(0.80)	3.04(0.92)	2.97(0.79)	2.83(0.78)
MA	4.58(0.85)	-	0.05	−0.11	0.03	−0.07	−0.06	0.08	−0.08
FA	4.14(1.05)	0.18 *	-	−0.04	0.02	−0.004	−0.08	−0.13	−0.01
Eros	3.85(0.74)	0.18 *	0.14	-	0.01	−0.38 ***	0.08	0.41 ***	0.41 ***
Ludus	2.47(0.77)	−0.15 *	−0.30 ***	−0.13	-	0.26 **	0.39 ***	−0.08	0.04
Storge	2.90(0.78)	−0.10	−0.22 **	0.01	0.28	-	0.31 ***	0.23 **	0.33 ***
Pragma	2.64(0.82)	−0.07	−0.13	−0.08	0.35 ***	0.28 ***	-	0.09	0.004
Mania	2.95(0.73)	−0.05	−0.04	0.17 *	0.22 **	0.41 ***	0.28 ***	-	0.27 ***
Agape	3.42(0.71)	−0.01	0.04	0.38 ***	−0.14	0.15 *	−0.08	0.38 ***	-

Note: *p* < 0.05 *; *p* < 0.01 **; *p* < 0.001 ***; MA = Mother Attachment; FA = Father Attachment.

## Data Availability

The data presented in this study are available on request from the corresponding author.
